# Disruption of tonic-clonic seizures using periodic stimulation of model neurons

**DOI:** 10.1186/1471-2202-12-S1-P11

**Published:** 2011-07-18

**Authors:** Bryce Beverlin, Theoden Netoff

**Affiliations:** 1 School of Physics and Astronomy, University of Minnesota, Minneapolis, MN 55455, USA; 2 Department of Biomedical Engineering, University of Minnesota, Minneapolis, MN 55455, USA

## 

Epileptic seizures change over time, arguably starting during the pre-ictal period progressing to the onset of the seizure, and then in most cases to some form of auto-termination. We show how periodic forcing, a model of deep brain stimulation (DBS), is able to change or suspend asynchronous dynamical states, effectively disrupting seizure activity [5]. In grandmal seizures, there is a “tonic” phase, where the patient will tense the muscles accompanied by high frequency low amplitude EEG, followed by a “clonic” phase, where the person has convulsive contraction of the muscles accompanied by low frequency large amplitude EEG. The underlying neuronal behaviors during these phase transitions are not completely understood. In pairwise recordings of neurons in brain slice models of seizures, it has been observed that synchrony, as measured by the precise timing of the synaptic inputs of two cells in close proximity to each other, decreases during the tonic phase of the seizure and then increases towards the end [4]. Thus, the transition from tonic to clonic phases may be caused by a change in synchrony in the neuronal network. Early disruption of the seizure is the goal of many clinical efforts using DBS.

We use a modified Morris-Lecar (ML) conductance based neuronal model [3] to demonstrate how the shift in seizure behavior from tonic phase to clonic phase can arise by modification of cell dynamics. During the tonic phase, we measure the response of perturbation in the ML oscillator cell at several phases to construct an input-output function called the phase resetting curve (PRC) [1]. Using the PRC we are able to conduct large scale network simulations of PRC model cells [2]. We then periodically drive the cells with a pulse to simulate DBS with various strengths and ratios of stimulus frequency to natural frequency of oscillation. A measure of entropy of the dispersion of cell phases provides information about the level of network synchrony. Dynamical changes in network synchrony resulting from periodic forcing indicate that DBS may be useful by changing cell population behavior. By examining a range of stimulation parameters we are able to predict which combinations of strength and frequency promote or prevent transition into seizures or between the tonic-clonic phases. These combinations are then used to stimulate the population of more biologically relevant conductance based model cells. By selecting proper combinations of strength and frequency of stimulation, we are able to control asynchronous states, which can lead to a suspension of the tonic phase until seizure termination. This work may be very useful to understand the underlying mechanisms of DBS already used in clinical applications and inform clinicians how to more effectively use DBS therapy on epileptic patients.
